# Supplementary material for: Facilitators, Barriers and Views on the Role of Public Health Institutes in Promoting and Using Health Impact Assessment—An International Virtual Scoping Survey and Expert Interviews
Source: Int J Environ Res Public Health. 2022 Oct 16;19(20):13367. doi: 10.3390/ijerph192013367 (PMC9602578; doi:10.3390/ijerph192013367)
Supplement: Supplementary file 1 [file ijerph-19-13367-s001.zip › ijerph-1927609-supplementary.pdf]

Supplementary Material S1 – Survey questionnaire.

Capturing the Social Value and Health Impact of Public Health Institutes Survey

Thank you for taking the time to complete this survey. It will take around 20 minutes to complete and all answers will be stored at a secure place, anonymised and only shared with the concerned research team.

Your participation is entirely voluntary, you do not have to answer every question and you are free to withdraw at any time. The first three sections of the survey focus on economic evaluation methodologies and social value. The final section of the survey focuses on Health Impact Assessment. Please answer all sections relevant to your role and expertise.

Q1. In what country is your Institute based?

Q2. Please state the name of your Institute *(optional)*

Q3. What focus does your Institute have? (*please select one answer option*)

National / Regional/ Other (please specify)

Q4. In what year was your Institute established?

Q5. Are the services or programmes provided or commissioned by your Institute, informed by any economic evaluation? (For example, cost-effectiveness or cost-benefit analysis (CEA/CBA), return on investment (ROI), etc.) *(please select one answer option)*

All of them / Some of them / None / Don’t know

If your institute use economic evaluation to inform their services or programmes:
a) Does this economic evaluation consider the physical/mental health impacts? *(please select one answer option)*

Yes, both / Yes, only the physical health impacts / Yes, only the mental health impacts / None / Don't know

b) Does this economic evaluation consider the social or community impacts? *(please select one answer option)*

Yes / No / Don't know

c) Does this economic evaluation consider the Environmental impacts? *(please select one answer option)*

Yes / No / Don't know

d) Does this economic evaluation consider the Economic impacts? *(please select one answer option)*

Yes / No / Don't know

Please provide more detail

Social Value

Social value is defined as the quantification of the relative importance that people place on the changes they experience in their lives accounting for the broader human and societal factors that result from an intervention or action. By asking individuals what has changed in their lives, it is possible to understand the wider results of our actions, not only on physical health, but also on social, economic and environmental factors.

Q6. Before being asked to complete this survey:

a) Had you heard about 'Social Value'? *(please select one answer option)*

Yes / No

b) Do you know what it means?

Yes / No

Q7. What do you think would be the benefits of capturing the Social Value of a Public Health programme? *(Please select all that apply)*

- Greater stakeholder engagement
- Capturing the social outcomes and impacts
- Capturing the environmental outcomes and impacts
- Quantifying and monetizing outcomes (showing their financial value)
- Being accountable to stakeholders
- Being accountable to funders
- Improved service design and delivery
- Enable organisations to act on the social determinants of health
- Make the case for investing in public health, based on evidence
- None
- Other (please specify)

Q8. What barriers do you think prevent Public Health Institutes from capturing their Social Value? *(Please select all that apply)*

- Not a priority at present (for the Institute or Government)
- Lack of awareness
- Lack of training
- Lack of resources

- Capacity
- None
- Other (please specify)

Q9. Does your Institute currently capture or measure the Social Value of the public health programmes provided/commissioned? *(please select one answer option)*

Yes / No / Don't know

Q9a). If Q9 = yes, was this work done in house or commissioned externally? *(please select one answer option)*

In house / Commissioned externally

Q9b). If Q9 = yes, do you know what methods are used to capture this*? (Please select all that apply)*

- Social Cost Benefit Analysis
- Social Return on Investment
- Other (please specify)

Q9c). If Q9 = yes, what impact do you think this has had?

Q10. Would your Institute like to do more to understand and measure the wider outcomes and impacts that public health programmes have? *(please select one answer option)*

Yes / No / Don't know

Q11. What do you think would help your Institute to capture (and measure) Social Value? *(Please select all that apply)*

- Specialist training
- Targeted resources to help increase awareness
- Targeted resources to help increase knowledge and skills (create capacity)
- Change in culture
- Examples of good practice
- Other (please specify)

Q12. Do you think capturing the Social Value of your Institute could contribute to a sustainable recovery from COVID-19 pandemic? *(please select one answer option)*

Yes, it should be a priority / Yes, but there are other priorities / No / Don't know

Please explain your answer

Supplementary Material S1 – Survey questionnaire.

Economic Evaluation and Social Return on Investment (SROI)

Q13. Are you aware of your Institute using health economics methods to measure (Social) Value and impact, for example cost-benefit analysis? *(please select one answer option)*

Yes / No / Don't know

Q14. Before completing this survey, were you aware of the 'Social Return on Investment (SROI)' method? *(please select one answer option)*

Yes / No

Q15. Are you aware of your Institute ever having used Social Return on Investment to capture and measure social value? *(please select one answer option)*

Yes, in the past but not anymore / Yes, occasionally when resources allow / Yes, frequently / No / Don't know

If yes, please describe:

Q16. Does your Institute currently advocate for more investment (resources) for public health or prevention? *(please select one answer option)*

Yes / No / Don't know

Q16a). If Q16 = yes, do you know what arguments/evidence is used to make the case for investment in public health/prevention? *(please select one answer option)*

Yes / No

If yes, please describe:

Q17. Does your Institute currently have a dedicated team/lead for using economic evaluation of public health programmes? *(please select one answer option)*

Yes / No / Don't know

Q18. What (if any) are the barriers to the use of economic evaluation, including measuring Social Value, in your Institute? ***(Please select all that apply)***

- Not an area of prioritisation at present
- Lack of knowledge
- Lack of training
- Lack of ability to advocate for it
- Lack of resources
- None

- Other (please specify)

Q19. How do you think awareness of the Social Value concept and the Social Return on Investment method could be improved?

Health Impact Assessments (HIA)

HIA is commonly defined as 'a combination of procedures, methods and tools by which a policy, programme or project may be judged as to its potential effects on the health of a population, and the distribution of those effects within a population'. The participatory nature of a HIA incorporates high levels of stakeholder engagement throughout the process, to account for the health, economic, social and environmental impacts of a policy, programme, service or project on health, well-being and population inequalities.

Q20. Before completing this survey, were you aware of the Health Impact Assessment method? *(please select one answer option)*

Yes / No

Q21. Does your Institute currently undertake Health Impact Assessments? *(please select one answer option)*

Yes / No / Don't know

Q21a). If Q21 = yes, please specify in which areas they are undertaken e.g. air quality, housing

Q21b). If Q21 = yes, does your Institute have a dedicated resource/team/lead for Health Impact Assessments? *(please select one answer option)*

Yes / No / Don't know

Q21c). If Q21 = yes, has your Institute developed a Health Impact Assessment guide or toolkit? *(please select one answer option)*

Yes / No / Don't know

Q21d). If Q21 = yes, are the Health Impact Assessments quality assured? *(please select one answer option)*

Yes / No / Don't know

Q22. Does your Institute currently advocate for using Health Impact Assessments in policy/decision-making? *(please select one answer option)*

Yes / No / Don't know

Q23. In your region or country, are you aware of any legislation/regulation which makes Health Impact Assessment mandatory? ? *(please select one answer option)*

Yes / No

Q23a). If Q23 = yes, for which fields of application? *(Please select all that apply)*

- Health policy
- Environment
- Others (please specify) [ ]

Q23b). If Q23 = yes, at which level? *(please select one answer option)*

National / Regional / Both

Q24. Does your Institute currently have a lead for Health in All Policies? *(please select one answer option)*

Health in All Policies is an approach to public policies across sectors that systematically takes into account the health implications of decisions, seeks synergies, and avoids harmful health impacts in order to improve population health and health equity.

Yes / No / Don't know

Q25. What (if any) are the barriers to the use of Health Impact Assessment in your Institute? *(Please select all that apply)*

- Not an area of prioritisation at present
- Lack of knowledge
- Lack of training
- Lack of ability to advocate for it
- Lack of resources
- None
- Other (please specify) [ ]

Q26. Would your Institute like to develop your knowledge and capacity around Health Impact Assessments? *(please select one answer option)*

Yes / No / Don't know

Q27. How do you think the effectiveness of Health Impact Assessments could be improved?

[ ]

Q28. How do you think awareness of Health Impact Assessment methodology could be improved?

Q29. How do you think awareness of Health Impact Assessment outputs could be improved?

Q30. Please share any other comments and reflections.

Thank you for taking the time to participate in this survey.

Would you be interested in participating in a semi-structured interview with us to provide further in-depth information to progress our work?

Yes / No

If yes, please can you provide your email address for us to contact you on:

When this work has been completed, we will be running a webinar to disseminate and share the results. If you are interested in attending please can you provide your email address:
